# Preoperative respiratory training with incentive spirometry for the prevention of pulmonary complications after liver surgery- a randomized pilot trial (PreSpi Trial)

**DOI:** 10.1007/s00423-025-03903-5

**Published:** 2025-10-21

**Authors:** Esther Giehl-Brown, Henriette Rangnick, Johannes Schweipert, Michael Halank, Dirk Koschel, Jürgen Weitz, Carina Riediger

**Affiliations:** 1 Department of Visceral, Thoracic and Vascular Surgery, University Hospital Carl Gustav Carus, Medical Faculty, Technische Universität Dresden, Fetscherstraße 74, 01307 Dresden, Germany; 2 National Center for Tumor Diseases (NCT/UCC), Dresden, Germany; 3 Department of General, Visceral and Transplantation Surgery, University of Heidelberg, Heidelberg, Germany; 4 Division of Pulmonology, Medical Department I, University Hospital Carl Gustav Carus, Technische Universität Dresden, Dresden, Germany; 5 Department of General and Visceral Surgery, Marienhospital Stuttgart, Böheimstraße 37, 70199 Stuttgart, Germany

**Keywords:** Randomized pilot trial, Preoperative respiratory training, Incentive spirometry, Postoperative pulmonary complications, Liver surgery

## Abstract

**Purpose:**

Postoperative pulmonary complications (PPCs) following liver surgery are associated with considerable morbidity and mortality. Nevertheless, data regarding the effectiveness of short-term, self-conducted preoperative respiratory training on pulmonary function and its influence on postoperative recovery are limited.

**Methods:**

Patients scheduled for liver surgery at the University Hospital Dresden, were screened for eligibility and randomized 1:1 to the intervention or control group. The intervention consisted of self-conducted respiratory training with an incentive spirometer for 14 consecutive days before surgery. Pulmonary function was assessed using longitudinal bodyplethysmography. The feasibility of the study design and clinical outcomes were evaluated.

**Results:**

Sixty-two patients were screened, 50 participants (81%) were randomized. Twenty control and 21 intervention participants (66.1%) completed all assessments and were included in the final analysis. Participants in the intervention group were highly compliant with the proposed inhalation training. Preoperative risk stratification for PPCs conducted according to the ARISCAT score revealed a high risk for PPCs in > 90% of all patients. Pneumonia occurred in 0 of intervention patients compared to 5 (25%) in the control group (*p* = 0.016). Pleural effusion was observed in 8 (38.1%) of intervention patients versus 16 (80%) in the control group (*p* = 0.007).

**Conclusion:**

This pilot trial establishes the feasibility of a definitive randomized controlled trial to investigate the effect of short-term, self-conducted preoperative respiratory training on pulmonary function for the prevention of PPCs after hepatic resection.

**Supplementary Information:**

The online version contains supplementary material available at 10.1007/s00423-025-03903-5.

## Introduction

Postoperative pulmonary complications (PPCs) are common after liver surgery and negatively impact postoperative recovery [1–3]. A PPC is any respiratory dysfunction that worsens the postoperative course [4]. According to the European Perioperative Clinical Outcome (EPCO) definition PPCs include respiratory infections, respiratory failure, pleural effusion, atelectasis, pneumothorax, bronchospasm, aspiration pneumonitis, pneumonia, acute respiratory distress syndrome (ARDS) and pulmonary embolus [5]. Reported incidences vary from 6.7% to 44.5% depending on patient population and procedure [1, 2], with severe PPCs occurring in up to 13% of patients after partial hepatectomy [3]. These complications substantially increase morbidity, mortality, and length of hospital stay [6, 7]. For example, pneumonia after colectomy raised 30-day mortality from 5.4% to 23.3% [7].

The risk of PPCs is linked to adverse effects of surgery and anesthesia on pulmonary function, including reduced end-expiratory muscle tone, loss of functional residual capacity, impaired ventilation–perfusion matching, and direct compression of lung tissue. Additional contributors are prolonged operative time, diabetes mellitus, and transverse subcostal incisions [1, 8, 9]. Several models aim to stratify PPC risk. The ARISCAT score, based on seven independent variables, divides patients into low-, intermediate-, and high-risk categories with corresponding PPC rates of 1.6%, 13.3%, and 42.1% [10–12]. While advanced age, incision type, and emergency surgery are non-modifiable factors, modifiable risks such as surgical duration and preoperative oxygenation may be improved by optimized anesthetic management and preconditioning of pulmonary function [13].

Preoperative inspiratory muscle training has been associated with fewer PPCs and shorter hospital stay in cardiac and abdominal surgery [14–16]. However, the quality of evidence in abdominal surgery is moderate to low, and no study to date has specifically examined its role in liver resection [17, 18]. Furthermore, the effect of respiratory training on perioperative pulmonary function remains to be clarified.

To address this evidence gap, we conducted the PreSpi randomized pilot trial to evaluate the feasibility and preliminary efficacy of a simple, self-administered preoperative respiratory training program in patients undergoing liver resection. This pilot study was designed to generate data on pulmonary function and clinical outcomes, thereby informing the design of a larger, definitive randomized controlled trial.

## Material and methods

### Study design

**Fig. 1:**
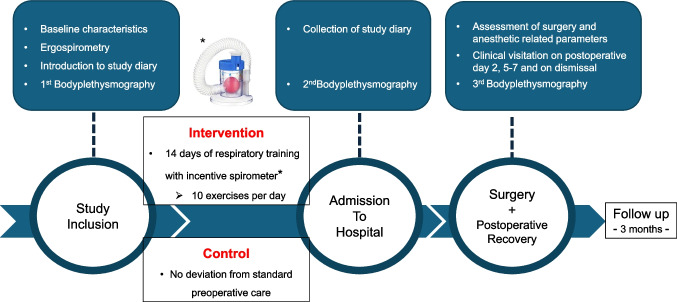
Flow chart of the study design. Patients scheduled for open liver resection were screened accordingly and provided written consent. Baseline ergospirometry and body plethysmography were performed. The intervention group received an incentive spirometer and training in daily breathing exercises, while the control group received no preoperative training. At hospital admission, study diaries were collected and body plethysmography was repeated. Surgery- and anesthesia-related parameters were recorded intraoperatively. Clinical assessments were performed on postoperative days 2, 5–7, and at discharge, with body plethysmography repeated before discharge. All patients underwent clinical follow-up at three months

This study has been designed as a randomized, monocentric pilot trial with two study arms: one intervention arm and one control arm. Patients scheduled for elective open liver surgery between August 2020 and April 2022 at the Department of Visceral, Thoracic and General Surgery, Technische Universität Dresden, Germany, were screened for participation. Block randomization with a fixed block size of five was performed, stratified according to minor and major hepatic resections. Randomization was conducted centrally by the study center in Dresden. Allocation concealment was ensured through the use of opaque, sequentially numbered, sealed envelopes prepared by an independent study coordinator who was not involved in patient recruitment or data collection. A total of 50 patients were randomized. The study intervention consisted of 14 consecutive days of respiratory training with an incentive spirometer before elective hospital admission (D_14_). At inclusion (D_0_), patients in the intervention arm were trained to perform inspiratory muscle exercises with the Mediflo Duo, a hand-held single-patient-use incentive spirometer with adjustable resistance. Patients were encouraged to perform 10 respiratory exercises a day, each consisting of 10 inspiratory exercises. The control arm was not exposed to any preoperative respiratory training. Baseline tests including patient characteristics, ergospirometry, bodyplethysmography, ARISCAT score, and laboratory findings were obtained from all patients at the time of study inclusion. Bodyplethysmography was repeated on the day before surgery (D_14_). Daily activity, compliance with respiratory training, and subjective respiratory improvement were assessed through an intervention diary for the intervention arm or a daily activity diary for the control arm. The postoperative course was monitored on days 2, 5, 7, and the day of dismissal. All data were documented in the corresponding case report forms (Fig. 1). All outcome assessors, including the clinical team evaluating postoperative outcomes and technicians performing functional testing (body plethysmography and ergospirometry), were blinded to group allocation.

### Inclusion criteria

Patients had to meet the inclusion criteria of age > 18 years, ASA (American Society of Anesthesiologists) Score I-III, and be scheduled for elective, open liver surgery under general anesthesia with protective lung ventilation. A minimum of 14 days between study inclusion and the day of surgery was required for the intervention. Written and oral informed consent from participants, as well as the ability and willingness to comply with the respiratory training, were mandatory.

### Exclusion criteria

Patients undergoing emergency surgery or those with mental disabilities or congestive heart failure > NYHA II were not eligible. Additionally, exclusion criteria included contraindications for open liver surgery, a history of pulmonary embolism and/or venous thrombosis within the previous 6 months, or previous pulmonary surgery. The proposed surgical procedure was limited to liver surgery, excluding multi-visceral resections, diaphragmatic resections, or ALPPS (associated liver partition and portal vein ligation for staged hepatectomy).

### Endpoint

The primary endpoint of the study was the incidence of postoperative pulmonary complications (PPCs), including acute respiratory distress syndrome (ARDS), pneumonia, aspiration pneumonia, airway infection, pleural effusion, atelectasis, pulmonary embolism, pneumothorax, and bronchospasm. The diagnosis was based on the European Perioperative Clinical Outcome (EPCO) definition [5].

Secondary endpoints of this trial included the influence of standardized preoperative respiratory training on preoperative and postoperative pulmonary function, differences in intraoperative blood gas analysis at the time of induction and emergence, differences in ventilation parameters (FiO_2_, PEEP), length of ventilation, length of ICU (intensive care unit) and hospital stay, occurrence of re-intubation, re-surgery or re-admission to the ICU, postsurgical interventions, postoperative morbidity, and mortality. Preoperative cardiopulmonary morbidity was stratified based on cardiopulmonary functional tests using ergospirometry combined with the Borg rating of perceived exertion. Additionally, the completeness, adherence to, and practicability of respiratory training were assessed through the intervention diary. Subgroup analyses, based on the randomization stratification, were also performed for major and minor liver resections.

### Statistical analysis

Statistical analysis was performed using IBM SPSS Statistics software, version 27 (SPSS Inc., Chicago, IL, USA). Since the study was a pilot trial, no formal sample size calculation was performed. Data are presented as means ± standard deviation or, where appropriate, as median values and interquartile range (IQR). Group comparisons were conducted using the Mann–Whitney U test for continuous variables and Pearson’s chi-squared test with Yates’ continuity correction for categorical variables. The two-sided Fisher’s exact test was used when the expected cell count in any cell of the chi-square test was below five. All statistical tests were conducted two-sided, and a p-value < 0.05 was considered statistically significant.

### Ethical aspects

The study was conducted in accordance with the Declaration of Helsinki with waivers of informed consent for all patients. Ethical approval from the local ethics committee was obtained before screening (Number: BO-EK-221052020). The trial was registered with the German Clinical Trials Register (Deutsches Register Klinische Studien (DRKS)/Nr.: DRKS00030325).

## Results

A total of 62 patients were screened for study inclusion, of whom 50 patients met inclusion criteria and were subsequently randomized. Two participants withdrew from the study, two participants were lost to follow-up and five participants had a change in treatment plan (see CONSORT 2010 Flow Diagram). These nine participants were excluded from the data analysis, and 41 participants (*n* = 21 in the intervention and *n* = 20 in the control arm) were included. Enrollment, allocation, follow-up, and analysis are depicted in the CONSORT 2010 flow diagram (Supplementary Fig. 1) and documented in detail in the CONSORT 2010 checklist (Supplementary Table 3) [19].

### Patient characteristics and surgical techniques

In the majority of cases, patients presented with ECOG performance status 1 (52.4% vs. 45% in the intervention and control group) or status 2 (42.9% vs. 40% in the intervention and control group). The most common comorbidities were cardiovascular diseases (57.1% in the intervention group vs. 75% in the control group) and previous surgeries were frequent (90.5% in the intervention group vs. 85% in the control group). According to the ARISCAT score, most patients were classified as high risk for PPCs (95.2% in the intervention group vs. 90% in the control group; Table 1). Despite randomization, patients in the intervention group (*n* = 21) were younger with a median age of 62.3 years, compared to the control group (*n* = 20) with a median age of 69.4 years (Table 1). Female participants were more common in the intervention group. The primary indication for liver surgery was malignancy in both groups (85.7% in the intervention group vs. 95% in the control group). Underlying conditions for hepatic surgery included primary liver cancers (hepatocellular carcinoma, intrahepatic cholangiocarcinoma), and liver metastases from colorectal, kidney, or pancreatic cancer or sarcoma. Only four patients had benign diseases including polycystic liver disease and hepatolithiasis (Table 1).

**Table 1:** Baseline characteristics of the intervention and control study cohorts

	Intervention Study cohort	Control Study cohort
Number of Patients [*n*]	21	20
Gender [*n* (%)]		
female	10 (47.6)	3 (15)
male	11 (52.4)	17 (85)
Age in years [median (min–max)]	62.3 (42–80)	69.4 (51–81)
BMI [kg/m^2^] [*n* (%)]		
Normal weight (18.5–24.9)	7 (33.3)	4 (20)
Overweight (25–29.9)	4 (19)	9 (45)
Obesity (> 29.9)	10 (47.6)	7 (35)
ASA *Physical Status Classification* [*n* (%)]		
2: mild systemic disease	8 (38.1)	5 (25)
3: severe systemic disease, not life threatening	13 (61.9)	15 (75)
ECOG performance status [*n* (%)]		
0 – asymptomatic	11 (52.4)	9 (45)
1 – symptomatic, fully ambulatory	9 (42.9)	8 (40)
2 – symptomatic, in bed < 50% of the day	1 (4.8)	3 (15)
ARISCAT Score [*n* (%)]		
26–44 points: Intermediate risk	1 (4.8)	2 (10)
45–123 points: High risk	20 (95.2)	18 (90)
Indication for surgery [*n* (%)]		
Malignant	18 (85.7)	19 (95)
- Hepatocellular cancer	2	7
- Intrahepatic cholangiocarcinoma	2	2
- Metastases		
• Colorectal cancer	12	7
• Others (including kidney, sarcoma, pancreas)	2	3
Benign	3 (14.3)	1 (5)
- Cystic disease (oligo-, polycystic)	2	1
- Hepatolithiasis	1	-
Pre-existing disease [*n* (%)]		
Cardiovascular disease	12 (57.1)	15 (75)
Pulmonary disease	4 (19)	3 (15)
- chronic obstructive pulmonary disease	0	1
- asthma	0	1
- obstructive sleep apnea syndrome	1	1
- pulmonary metastases	1	1
- pulmonary embolism	1	-
Gastrointestinal disease	9 (42.9)	12 (60)
Endocrine disease	8 (38.1)	16 (80)
Previous surgeries	19 (90.5)	17 (85)
Regular alcohol consumption [*n* (%)]		
- None	9 (42.9)	10 (50)
- Occasional	11 (52.4)	6 (30)
- Regular	1 (4.8)	4 (20)
Nicotine abuse in pack years [*n* (%)]		
< 20	3 (14.3)	6 (30)
> 20	1 (4.8)	2 (10)
Allergies [*n* (%)]	14 (66.7)	15 (75)
Daily physical activity		
- Low (only daily chores)	6 (28.6)	8 (40)
- Intermediate (e.g. walking, gardening, biking)	14 (66.7)	10 (50)
- High (sporting activity, physical work)	1 (4.8)	2 (10)
Preoperative anemia (Hemoglobin < 8 mmol/L) [n (%)]	5 (23.8)	7 (35.0)

Abbreviations: *BMI* Body Mass Index, *ASA* American Society of Anesthesiologists, *ARISCAT* Assess Respiratory Risk in Surgical Patients in Catalonia, *n* number, *ECOG* Eastern Co-operative Oncology Group

Minor liver resections were performed in 20 patients (10 in the control group and 10 in the intervention group) including atypical and anatomical liver segment resection of up to 2 liver segments (Table 2). Major hepatectomies of more than 3 liver segments were performed in 21 patients (10 in the control group and 11 in the intervention group). The Makuuchi incision was most commonly used (90%, Table 2). The duration of the surgical procedure exceeded 3 h in 76% of participants, with no significant differences observed in intraoperative blood loss or the number of blood transfusions (Table 2). No intraoperative surgical or anesthetic complications were reported.

**Table 2:** Intraoperative parameters of the intervention and control study cohorts

	Intervention Study cohort *n* = 21	Control Study cohort *n* = 20	*p*-value
Abdominal incision [*n* (%)]			
- Makuuchi	19 (93.2)	17 (85)	.239
- Upper abdominal	1 (4.8)	2 (10)	
- Midline	0	1 (5)	
Surgical procedure* [*n* (%)]			
- Atypical liver resection	14 (66.6)	10 (50)	.225
- Anatomical liver resection	9 (42.8)	9 (45)	.168
- Hemihepatectomy			.514
- left	2 (9.5)	1 (5)	
- right	1 (4.8)	2 (10)	
- extended right	2 (9.5)	1 (5)	
Inferior Vena Cava Clamping [*n* (%)]	2 (9.5)	3 (15)	.841
Pringle maneuver [n (%)]	6 (28.6)	7 (35)	.448
Hb (mmol/L), before surgery (mean ± SD)	8.72 ± 0.98	7.84 ± 1.39	.022
Hb, after surgery (mean ± SD)	7.21 ± 0.87	6.77 ± 0.98	.132
Hct (%), before surgery (mean ± SD)	0.409 ± 0.04	0.376 ± 0.07	.057
Hct (%), after surgery (mean ± SD)	0.34 ± 0.04	0.32 ± 0.05	.259
Length of surgical procedure [*n* (%)]			.702
- < 180 min	5 (23.8)	5 (25)	
- 180–360 min	13 (61.9)	12 (60)	
- > 360 min	3 (14.3)	3 (15)	
Median intraoperative Positive End-Expiratory Pressure (in cm H_2_O) [*n* (%)]			.002
- 4 - 5	17 (81)	7 (35)	
- 6 - 7	2 (9.5)	6 (30)	
- > 7	2 (9.5)	7 (35)	
Median intraoperative inspired oxygen fraction (FiO2) [*n* (%)]			.347
- 0.35 - 0.40	4 (19)	6 (30)	
- 0.45 - 0.50	15 (71.4)	11 (55)	
- > 0.50	2 (9.5)	3 (15)	
Immediate extubation [*n* (%)]	19 (93.2)	16 (80)	.355
Total intraoperative blood loss [*n* (%)]			.540
< 500 ml	10 (47.6)	8 (40)	
< 1 L	5 (23.8)	9 (45)	
< 2 L	5 (23.8)	2 (10)	
> 2 L	1 (4.8)	1 (5)	
Intraoperative blood transfusions [(%)]			.625
- 1	0	2 (10)	
- 2	1 (4.8)	1 (5)	
- > 2	1 (4.8)	1 (5)	
Postoperative admission to [*n* (%)]			.633
- intensive care unit	11 (52.4)	12 (60)	
- surgical ward	10 (47.6)	8 (40)	

* Multiple resection techniques per patients were applied where applicable

Abbreviations: *Hb* Hemoglobin, *Hct* Hematocrit

### Adherence to the intervention and activity level

In the intervention group, adherence to the breathing exercises was monitored through an intervention diary and patient questionnaire (Supplementary Table 1). Overall, patients demonstrated high compliance with the proposed inhalation training, performing an average of 9.4 exercises per day, each consisting of 9.8 breaths. This closely matched the goal of 10 exercises a day, with 10 inspirations per exercise. Of the 21 patients, 15 reported experiencing a benefit with the training. In the control group, the activity diary indicated a similar level of physical activity 14 days before surgery. Most patients in both groups engaged in only daily chores; a few exercised lightly, such as walking their dog or tending their garden, while only a minority performed strenuous exercise.

### Ergospirometry/borg score

All study participants underwent a cardiopulmonary exercise test. Ergospirometry parameters revealed no significant differences in cardiopulmonary function between groups. Additionally, there were no significant differences regarding the Borg rating of perceived exertion (Supplementary Table 2).

### Bodyplethysmography

Pulmonary function assessed by bodyplethysmography at the time point of inclusion showed no significant differences between the study cohorts (Table 3). Evaluation by bodyplethysmography was repeated on the day of admission for surgery.

**Table 3:** Body plethysmography results at baseline (D0) and after 14 days (D14) of respiratory training in the intervention group or at hospital admission (D14) in the control group

	Intervention study cohort			Control study cohort			*p*-value	*p*-value	*p*-value
	Inclusion (D_0_) [mean ± SD]	Admission (D_14_) [mean ± SD]	D_0_-D_14_ [mean ± SD]	Inclusion (D_0_) [mean ± SD]	Admission (D_14_) [mean ± SD]	D_0_-D_14_ [mean ± SD]	Inclusion (D_0_)	Admission (D_14_)	D_0_-D_14_
Patients [n]	21	21		20	20				
FVC (L)	3.82 ± 0.9	3.77 ± 1.0	0.002 ± 0.20	3.45 ± 1.0	3.45 ± 1.1	0.002 ± 0.21	.239	.259	.260
VC IN (L)	3.78 ± 0.97	3.85 ± 0.95	**- 0.070 ± 0.12**	3.55 ± 0.95	3.51 ± 0.98	**0.045 ± 0.21**	.449	.261	**.041**
IC (L)	2.87 ± 0.82	2.99 ± 0.86	- 0.123 ± 0.19	2.68 ± 0.68	2.67 ± 0.69	0.002 ± 0.14	.417	.199	.127
ERV (L)	0.97 ± 0.38	0.90 ± 0.32	0.062 ± 0.18	0.89 ± 0.44	0.85 ± 0.45	0.045 ± 0.14	.573	.657	.095
Residual volume (L)	2.33 ± 0.52	**2.24 ± 0.46**	0.097 ± 0.15	2.73 ± 0.89	**2.70 ± 0.81**	0.032 ± 0.20	.096	**.032**	.319
RV%TLC (%)	38.4 ± 8.3	**36.8 ± 7.9**	1.585 ± 1.97	43.1 ± 8.6	**43.3 ± 8.6**	- 0.216 ± 1.67	.084	**.016**	.160
FEV_1_ (L)	2.87 ± 0.7	2.81 ± 0.7	0.021 ± 0.12	2.58 ± 0.8	2.53 ± 0.8	0.049 ± 0.19	.251	.212	.335
Tiffeneau-Index (%)	75.1 ± 7.0	74.9 ± 6.8	0.250 ± 2.58	74.6 ± 8.2	73.2 ± 8.1	1.410 ± 3.70	.847	.492	.270
Peak flow (L/s)	7.82 ± 2.1	7.6 ± 1.9	0.091 ± 0.52	7.75 ± 2.3	7.6 ± 2.4	0.155 ± 0.74	.913	.834	.270
MEF at 25% FVC (L/s)	0.86 ± 0.40	**0.86 ± 0.33**	0.000 ± 0.26	0.69 ± 0.26	**0.65 ± 0.32**	0.047 ± 0.13	.117	**.041**	.459
MEF at 50% FVC (L/s)	3.13 ± 1.4	3.0 ± 1.2	0.091 ± 0.59	2.67 ± 1.1	2.63 ± 1.2	0.040 ± 0.34	.239	.269	.388
MEF at 75% FVC (L/s)	5.97 ± 2.1	5.9 ± 2.1	0.027 ± 0.44	5.82 ± 2.3	6.00 ± 2.4	- 0.176 ± 0.92	.830	.939	.232
Airway resistance (kPa/(L/s))	0.30 ± 0.1	0.30 ± 0.1	0.000 ± 0.08	0.34 ± 0.2	0.31 ± 0.1	0.021 ± 0.07	.455	.732	.237

*P*-values for within-group changes (D0–D14) and between-group differences are shown

Abbreviations: *n* Number, *FVC* forced vital capacity, *VC IN* inspiratory vital capacity, *IC* inspiratory capacity, *ERV* expiratory reserve volume, *RV%TLC* residual volume expressed as percent of total lung capacity, *FEV1* volume that has been exhaled at the end of the first second of forced expiration, *MEF* maximal endexpiratory flow. Note: *[mean ± SD]

The intervention group exhibited significantly lower residual volumes (2.24L vs. 2.70L; *p* = 0.032; Table 3) and residual volumes as a percentage of total lung capacity (36.8% vs. 43.3%; *p* = 0.016; Table 3) as well as increased maximal endexpiratory flows at 25% of forced vital capacity (0.86L/s vs. 0.65L/s; *p* = 0.041; Table 3), compared to the control group. Additionally, the increase in inspiratory vital capacity before and after training was significant in the intervention group, whereas the control group showed a slight decrease (0.070L vs. −0.045L; *p* = 0.041; Table 3). No differences were observed between the two groups in the postoperative recovery period when comparing bodyplethysmography results (data not shown). Importantly, abdominal incisions limited inspiratory capacity due to wound pain, which may have reduced the validity of these results.

### Postoperative outcomes

Postoperative mortality was comparable between the intervention and control cohorts (4.8% vs. 5.0%, respectively). According to the Clavien–Dindo classification, most complications were grade I–II (9/21 in the intervention cohort (42.9%) vs. 9/20 in the control cohort (45.0%); Table 4). Complications requiring intervention under local anesthesia (Clavien–Dindo IIIa), such as image-guided percutaneous drainage, occurred in 2/21 (9.5%) in the intervention cohort and 5/20 (25.0%) in the control cohort. Additionally, 2/20 (10.0%) control patients, but no intervention patients, required reintervention under general anesthesia (Clavien–Dindo IIIb). Overall, complication rates and severity were reduced in the intervention group compared with controls (*p* = 0.042).

**Table 4:** Postoperative outcomes of the intervention and control groups

	Intervention Study cohort *n* = 21	Control Study cohort *n* = 20	*p*- value
Length of hospital stay in days (mean ± SD)	10.4 ± 5.1	14.9 ± 10.0	.086
Length of ICU stay in days (mean ± SD)	0.70 ± 0.801	1.00 ± 1.556	.450
PPC [*n* (%)]	11 (52.4)	16 (80)	.062
Pneumonia [*n* (%)]			**.016**
- Dindo I	-	-	
- Dindo II	-	4 (20)	
- Dindo V	-	1 (5)	
Respiratory failure [*n* (%)]			.141
- Dindo II	-	1 (5)	
- Dindo IIIa	-	2 (10)	
- Dindo V	1 (4.8)	1 (5)	
Pleural effusion [*n* (%)]			**.007**
- Dindo I	5 (23.8)	7 (35)	
- Dindo II	1 (4.8)	5 (25)	
- Dindo IIIa	2 (9.5)	4 (20)	
Atelectasis [*n* (%)]			.084
- Dindo I	6 (28.6)	6 (30)	
- Dindo II	1 (4.8)	5 (25)	
- Dindo IIIa	2 (9.5)	3 (15)	
Pulmonary embolism [*n* (%)]			.092
- Dindo II	5 (23.8)	1 (5)	
Surgical complications [n (%)]			**.042**
- Dindo I	3 (14.3)	4 (20)	
- Dindo II	6 (28.6)	5 (25)	
- Dindo III	2 (9.5)	5 (25)	
- Dindo IV	-	2 (10)	
- Dindo V	1 (4.8)	1 (5)	

Abbreviations: n number, ICU intensive care unit, PPC postoperative pulmonary complications, Dindo Clavien-Dindo classification of surgical complications

The incidence of PPCs was lower in the intervention group (11/21 (52.4%)) compared to the control group (16/20 (80.0%); *p* = 0.062; Table 4). Pneumonia occurred in 0/21 (0%) of intervention patients versus 5/20 (25.0%) of controls (*p* = 0.016). Pleural effusion was observed in 8/21 (38.1%) of intervention patients compared with 16/20 (80.0%) of control patients (*p* = 0.007). Rates of atelectasis (9/21 (42.9%) vs. 14/20 (70.0%); *p* = 0.084) and respiratory failure (1/21 (4.8%) vs. 4/20 (20.0%); *p* = 0.141) did not differ significantly between intervention and control groups, respectively. No cases of tracheobronchitis, bronchospasm, ARDS, or pulmonary edema occurred in either group. Notably, pulmonary embolism was more frequent in the intervention group (5/21 (23.8%)) compared with the control group (1/20 (5.0%); *p* = 0.092; Table 4).

## Discussion

Postoperative pulmonary complications (PPCs) are common in patients undergoing hepatic resection, contributing to increased overall morbidity, mortality, and prolonged hospital stay [1, 3, 6, 20]. While various perioperative intervention strategies have been explored to reduce the risk for PPCs, preoperative intervention approaches are limited, and conclusive evidence of efficacy is lacking [21]. The results of this pilot study highlight the feasibility of a future definitive randomized controlled trial (RCT) by evaluating the effect of short-term, self-conducted preoperative respiratory training on pulmonary function and its prevention of PPCs after hepatic resection.

The observed recruitment rate (81%) was sufficient to enroll 50 participants within 20 months at a German University hospital. The most frequent reason for participants to decline was the additional time and physical efforts of ergospirometry and bodyplethysmography. Ergospirometry was performed at inclusion to assess the cardiorespiratory function of study participants to ensure comparability at inclusion. Accounting for alternative ways to eliminate relevant bias related to baseline differences should be anticipated. During the study, 9 of 50 participants had to be withdrawn from the trial (18%). 5 participants encountered a change in their treatment plan, causing a rescheduling of surgery or a deviation of the surgical procedure due to intraoperative findings. This relatively high drop-out rate can be attributed to the predominantly oncologic nature of the study population. Nevertheless, we believe that this patient cohort benefits from preventive PPC measures as multimodal treatment plans rely on a smooth postoperative recovery.

Within the whole study cohort, the occurrence of PPCs (pleural effusions (58%), atelectasis (56%), pneumonia (12%), respiratory failure (12%), and pulmonary embolism (14.6%) were frequent, emphasizing the relevance of this study. Reported frequencies are also comparable with the literature [1, 3, 6, 20]. The present study suggests that postoperative pneumonia and pleural effusions were reduced in the intervention group (*p* = 0.007 and *p* = 0.016, respectively). Preoperative respiratory training was associated with reduced surgical complications overall (*p* = 0.042).

An unexpected finding of this study was the higher incidence of pulmonary embolism in the intervention group (23.8%) in comparison to controls (5.0%). Given the limited sample size and pilot nature of the trial, this difference should be interpreted with caution and may represent an incidental finding. All patients received standard perioperative thromboprophylaxis, and no established pathophysiological mechanism links incentive spirometry to an increased risk of venous thromboembolism. Nonetheless, it cannot be excluded that altered intrathoracic pressure dynamics or differences in mobilization may have contributed in predisposed patients. Larger, adequately powered studies will be required to clarify whether this reflects random variation or a true association.

While some outcomes did not reach statistical significance, several trends may hold clinical relevance. For example, the lower rate of postoperative pulmonary complications in the intervention group (52.4% vs. 80.0%, *p* = 0.062) suggests a meaningful effect size despite limited statistical power. Similarly, reductions in atelectasis and respiratory failure did not achieve statistical significance but may still be clinically important. These results should therefore be considered hypothesis-generating and warrant confirmation in a larger randomized trial.

The preventive effect of preoperative inspiratory muscle training on the reduction of Clavien-Dindo Class I postoperative complications after lung resection has been described previously [22]. However, recommendations drawn from this study cannot be easily transferred to patients undergoing abdominal or especially hepatic surgery. This is due to differences in preoperative patient characteristics as well as surgical and anesthesiologic procedures in thoracic surgery. This highlights the need for a definitive RCT in hepatobiliary surgery.

Evaluation of patients’ basic characteristics revealed a frequent presence of risk factors for PPCs including advanced age and obesity [13]. Block randomization was stratified according to only the extent of resection. Additional known risk factors for PPCs should be stratified in a future RCT to ensure similar entry characteristics. Liver surgery was associated with procedure-related risk factors for PPCs including upper abdominal surgical incision, duration of surgery above 3 h, and requirement of general anesthesia. This was reflected by high ARISCAT scores (≥ 90% in both groups). The cardiorespiratory function was comparable between groups at the point of inclusion according to ergo spirometry results. General physical activity levels did not differ between both groups during the 14 days before hospital admission. Patients in the intervention group reported high compliance with the proposed respiratory training using incentive spirometry. This underlines the participant’s willingness to commit to preventive measures to improve their health and the feasibility of the proposed training for a broader study population. Only 2 of the 26 participants allocated to the intervention group (7.7%) withdrew from the study because they were unable to perform respiratory training.

In contrast to our results, Kulkarni et al. did not observe any effect on PPCs. They compared different training approaches, which included utilizing an incentive spirometer in patients undergoing general abdominal surgery. However, the authors reported improved inspiratory muscle strength after preoperative inspiratory muscle training [15, 23]. Similar to our results, Boden et al. reported a 48% reduction in the incidence of PPCs within 14 days after surgery. This differed from our approach since the authors used one-time preoperative physiotherapy education and breathing exercise training in 441 adult patients undergoing elective open upper abdominal surgery [24]. Although the median age between 60 and 70 years and an ASA score of 1 or 2 in the majority of cases of the study population is comparable to our cohort, only 7% of patients underwent hepatic surgery in this trial [24]. Additionally, no assessment of pulmonary function was conducted and investigators focused primarily on postoperative mobilization [24]. These findings are coherent with earlier studies. They observed an association of preoperative patient counseling on postoperative mobilization and preoperative chest physiotherapy with a reduced risk for PPCs [25, 26].

In contrast, we provide a short-term, simple, and cost-effective training protocol that patients can independently perform before surgery, which we suggest is superior to one-time educational or physiotherapy sessions. Our results suggest furthermore that pulmonary function improved after respiratory training with incentive spirometry. Patients of the intervention cohort showed an increase in inspiratory vital capacity accompanied by a reduction in residual volume. Values reached significance despite the small size of the study population. We suggest that a decrease in residual volume and total lung volume is equivalent to a reduction in physiologic dead space. This differs from other studies, as we selected body plethysmography as functional testing to monitor changes in pulmonary function rather than focusing on inspiratory muscle strength. Previous studies have shown an association between reduced functional residual capacity after abdominal surgery and a predisposition for pulmonary complications [27, 28]. An increase in resting physiological dead space has been suggested as a potential predictor of PPCs after robotic-assisted lung resection [29]. An elevated physiologic dead space has been identified as a prognostic marker in acute respiratory distress syndrome, but clinical investigations in the area of abdominal surgery remain limited [30].

The main limitation of this study is its small cohort size, inherent to its pilot design. The single-center setting further restricts the generalizability of the findings, and the absence of prior data on preoperative respiratory training in hepatic surgery precluded a formal sample size calculation. Consequently, the trial was not powered to detect all clinically relevant differences, and some outcomes may represent random variation. These limitations underline that the results should be interpreted as exploratory and hypothesis-generating. Nevertheless, the study demonstrates feasibility and provides preliminary data to inform the design of a larger, adequately powered multicenter randomized controlled trial.

In conclusion, our results indicate the feasibility of a definite RCT to assess the impact of short-term, self-conducted preoperative respiratory training on pulmonary function, while monitoring changes via body plethysmography, on the occurrence of PPCs after hepatic resection. Furthermore, it suggests that preoperative respiratory training leads to improved pulmonary function and a reduced incidence of pleural effusions and pneumonia following open hepatic surgery.

## Supplementary Information

Below is the link to the electronic supplementary material.

Supplementary file1 (DOC 217 KB)

Supplementary file2 (DOC 51 KB)

Supplementary file3 (DOCX 16 KB)

Supplementary file4 (DOCX 19 KB)

## Data Availability

The data that supports the findings of this study are available on request from the corresponding author. The data are not publicly available due to containing information that could compromise the privacy of research participants.

## Declarations

### Clinical trial registration

The trial is registered in the German Clinical Trials Register (Deutsches Register Klinische Studien (DRKS)/Nr.: DRKS00030325).
