# Supplementary material for: Preoperative respiratory training with incentive spirometry for the prevention of pulmonary complications after liver surgery- a randomized pilot trial (PreSpi Trial)
Source: Langenbecks Arch Surg. 2025 Oct 21;410(1):306. doi: 10.1007/s00423-025-03903-5 (PMC12540556; doi:10.1007/s00423-025-03903-5)
Supplement: Supplementary file 2 — Supplementary file2 (DOC 51 KB) [file 423_2025_3903_MOESM2_ESM.doc]

**
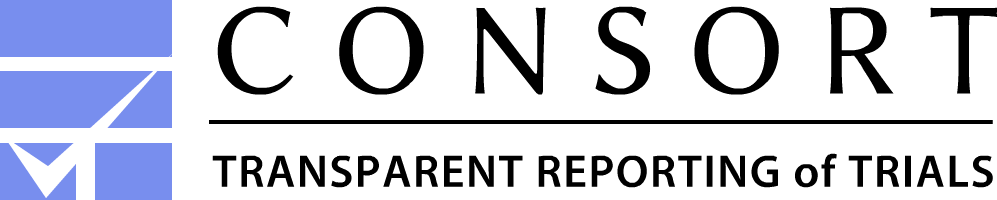
**

**CONSORT 2010 Flow Diagram**

**Allocation**

**Analysis**

**Follow-Up**

**Enrollment**

Assessed for eligibility (n= 62 )

Excluded (n= 12 )

♦  Not meeting inclusion criteria (n= 5 )

♦  Declined to participate (n= 6 )

♦  Other reasons (n= 1 )

Analyzed (n= 20 )
♦ Excluded from analysis (n= 4 )

Followed-up (n=20)

Lost to follow-up (n=4)

(unable to participate in pulmonary function tests: n= 1;

change of surgical procedure: n= 3)

Allocated to control (n= 24 )

♦ Received allocated intervention (n= 24 )

♦ Did not receive allocated intervention (n= 0 )

Followed-up (n=21)

Lost to follow-up (n=3)

(allocated to different hospital: n= 1;

change of surgical procedure: n= 2)

Allocated to intervention (n= 26 )

♦ Received allocated intervention (n= 24 )

♦ Did not receive allocated intervention (withdrawal from treatment: n= 2 )

Analyzed (n= 21 )
♦ Excluded from analysis (n= 5 )

Randomized (n= 50)
