# Supplementary material for: Preoperative respiratory training with incentive spirometry for the prevention of pulmonary complications after liver surgery- a randomized pilot trial (PreSpi Trial)
Source: Langenbecks Arch Surg. 2025 Oct 21;410(1):306. doi: 10.1007/s00423-025-03903-5 (PMC12540556; doi:10.1007/s00423-025-03903-5)
Supplement: Supplementary file 3 — Supplementary file3 (DOCX 16 KB) [file 423_2025_3903_MOESM3_ESM.docx]

|  | Intervention  Study Cohort | Control  Study Cohort | p- value |
| --- | --- | --- | --- |
| Patients [n] | 21 | 20 |  |
| Number of breathing exercises per day (goal=10) [mean (SD)] | 9.4 (1.5) | - | - |
| Number of breaths per exercise (goal=10) [mean (SD)] | 9.8 (0.5) | - | - |
| Physical activity 14 days before surgery [n (%)]   - Only daily activities (buying groceries, cooking, cleaning, etc.) - Light exercise (walking, biking, gardening, etc.) - Strenuous exercise | 13 (62)  6 (28)  2 (9.5) | 14 (70)  5 (25)  1 (5) | 0.80 |
| Motivation during training [n (%)]   - High - Mediocre - Low | 11 (52.4)  10 (47.6)  - | -  -  - | - |
| Subjective implementation of training [n (%)] | 21 (100) |  |  |
| Subjective success of training [n (%)] | 15 (71.4) | - | - |

**Supplementary table 1:** Parameters recorded in patients’ intervention (intervention group) and daily activity diary (control group; grey) respectively
