# Supplementary material for: Preoperative respiratory training with incentive spirometry for the prevention of pulmonary complications after liver surgery- a randomized pilot trial (PreSpi Trial)
Source: Langenbecks Arch Surg. 2025 Oct 21;410(1):306. doi: 10.1007/s00423-025-03903-5 (PMC12540556; doi:10.1007/s00423-025-03903-5)
Supplement: Supplementary file 4 — Supplementary file4 (DOCX 19 KB) [file 423_2025_3903_MOESM4_ESM.docx]

|  | Intervention  Study Cohort | Control  Study Cohort | *p* |
| --- | --- | --- | --- |
| Patients [n] | 21 | 20 |  |
| VO_2_ [mean (SD)]   - peak (ml*kg^-1^KG) - peak % Soll - at VT1 %Soll | 18.5 (5.8)  84.0 (20)  60.0 (12) | 18.1 (5.8)  82.5 (24)  65.5 (20) | .262  .210  .456 |
| Strength (watt) [mean (SD)]   - maximum - maximum %Soll | 108.0 (21)  72.0 (23) | 106.5 (52)  73.5 (29) | .696  .499 |
| VO_2_/watt ((ml/min)/W) [mean (SD)] | 11.0 (1.0) | 11.1 (1.0) | .989 |
| Heart rate [mean (SD)]   - maximum - max. %Soll | 146 (38)  97.0 (20) | 135 (36)  91.5 (30) | .074  .127 |
| Oxygen pulse (ml/beats) [mean (SD)] | 11.0 (4.7) | 13.4 (5.1) | .334 |
| Systolic blood pressure (mmHg) [mean (SD)]   - at rest - maximal exertion - normalization during recovery (n) | 122.0 (24)  177 (66)  19 | 119.0 (15)  176 (44)  20 | .714  .686  .162 |
| Breathing reserve [mean (SD)]   - (L/min) - % Soll | 47.0 (39)  43.0 (19) | 39.5 (32)  39.5 (18) | .223  .217 |
| Arterial oxygen pressure (paO2, kPa) [mean (SD)]   - at rest - maximal exertion | 11.3 (1.4)  12.9 (1.6) | 10.5 (1.7)  11.75 (1.8) | .057  .048 |
| Arterial carbon dioxide pressure (paCO2, kPa) [mean (SD)]   - at rest - maximal exertion | 4.59 (1.0)  4.71 (1.0) | 4.70 (0.6)  4.68 (0.9) | .860  .525 |
| Alveolar-arterial oxygen pressure difference  (AaDO2 in kPa) [mean (SD)]   - at rest - at maximal exertion | 2.35 (1.4)  2.87 (1.2) | 2.98 (1.4)  3.07 (1.0) | .256  .088 |
| End-tidal carbon dioxide partial pressure (PET-CO2) [mean (SD)]   - at rest - at VT1 - maximal | 3.87 (0.9)  5.01 (0.8)  4.52 (0.9) | 3.77 (0.5)  4.58 (0.8)  4.36 (0.7) | .797  .182  .507 |
| Capillary end-tidal carbon dioxide pressure  (P(kap-ET)CO2) [mean (SD)] | 1.57 (2.0) | 2.77 (2.5) | .014 |
| Ventilatory equivalent for oxygen EQO2 [mean (SD)]   - at rest - at AT1 - at maximal exertion | 29.50 (10.1)  26.70 (4.9)  38.60 (8.9) | 29.35 (6.4)  28.95 (4.5)  36.30 (7.6) | .904  .127  .738 |
| Ventilatory equivalent for CO2 (EQCO2) [mean (SD)]   - at rest - at AT1 - at maximal exertion | 36.8 (6.8)  28.90 (6.0)  32.1 (10.2) | 37.6 (4.0)  32.6 (5.0)  34.1 (7.5) | .341  .086  .310 |
| Ventilator efficiency (VE/VCO2) Slope [mean (SD)] | 28.9 (2.5) | 30.9 (5.8) | .179 |
| Borg rating – muscular exertion [n (%)]   - 0 – 2 (really) easy - 3 – 4 moderate - 5 – 6 hard - 7 – 8 really hard - 9 – 10 maximal | 3 (14.3)  5 (23.8)  8 (38.1)  4 (19.1)  1 (4.8) | 3 (15)  3 (15)  9 (45)  4 (20)  1 (5) | .905 |
| Borg rating – dyspnea [n (%)]   - 0 – 2 (really) easy - 3 – 4 moderate - 5 – 6 hard - 7 – 8 really hard | 4 (19.1)  10 (47.6)  6 (28.6)  1 (4.8) | 5 (20)  9 (45)  3 (15)  3 (15) | .671 |

**Supplementary table 2:** Results of preoperative ergo spirometry and BORG score

*L* liter, *min* minutes, *VO_2_* oxygen uptake, *KG* body weight, *SD* standard deviation, *VT1* ventilatory threshold.
